# An Attempt to Boost Posterior Population Expansion Using Fast Machine Learning Algorithms

**DOI:** 10.3389/frai.2021.624629

**Published:** 2021-03-18

**Authors:** Przemysław Juda, Philippe Renard

**Affiliations:** ^1^Stochastic Hydrogeology and Geostatistics Group, Centre for Hydrogeology and Geothermics, University of Neuchâtel, Neuchâtel, Switzerland; ^2^Department of Geosciences, University of Oslo, Oslo, Norway

**Keywords:** hydrogeology, inverse problem, posterior population expansion, binary classification, geostatistics, groundwater flow and transport, deep learning, ensemble learning

## Abstract

In hydrogeology, inverse techniques have become indispensable to characterize subsurface parameters and their uncertainty. When modeling heterogeneous, geologically realistic discrete model spaces, such as categorical fields, Monte Carlo methods are needed to properly sample the solution space. Inversion algorithms use a forward operator, such as a numerical groundwater solver. The forward operator often represents the bottleneck for the high computational cost of the Monte Carlo sampling schemes. Even if efficient sampling methods (for example Posterior Population Expansion, PoPEx) have been developed, they need significant computing resources. It is therefore desirable to speed up such methods. As only a few models generated by the sampler have a significant likelihood, we propose to predict the significance of generated models by means of machine learning. Only models labeled as significant are passed to the forward solver, otherwise, they are rejected. This work compares the performance of AdaBoost, Random Forest, and convolutional neural network as classifiers integrated with the PoPEx framework. During initial iterations of the algorithm, the forward solver is always executed and subsurface models along with the likelihoods are stored. Then, the machine learning schemes are trained on the available data. We demonstrate the technique using a simulation of a tracer test in a fluvial aquifer. The geology is modeled by the multiple-point statistical approach, the field contains four geological facies, with associated permeability, porosity, and specific storage values. MODFLOW is used for groundwater flow and transport simulation. The solution of the inverse problem is used to estimate the 10 days protection zone around the pumping well. The estimated speed-ups with Random Forest and AdaBoost were higher than with the convolutional neural network. To validate the approach, computing times of inversion without and with machine learning schemes were computed and the error against the reference solution was calculated. For the same mean error, accelerated PoPEx achieved a speed-up rate of up to 2 with respect to the standard PoPEx.

## 1. Introduction

Groundwater flow and contaminant transport in aquifers depend on subsurface parameters such as permeability, specific storage, or porosity. Due to the lack of their direct measurements and heterogeneity, solving inverse problem is essential to make reliable predictions of groundwater flow and crucial for water resources management.

The inverse problem consists in deducing the subsurface parameters given state variables measured in the field, such as hydraulic heads or tracer concentrations. The physical problem leading from parameters to state variables is called the forward problem; it often involves solving partial differential equations. While the forward problem has a unique solution, the inverse problem is usually ill-posed, with non-unique and unstable solution if framed in a deterministic manner. The inverse problem is especially difficult when dealing with highly heterogeneous parameter fields or categorical fields. Therefore, methods for solving the inverse problem in hydrogeology (and more broadly in geophysics) have been a topic of extensive research (Zhou et al., 2014; Linde et al., 2015). If defined in a probabilistic manner, the inverse problem is no longer ill-posed and the solution always exists (Tarantola, 2005). When formulated in a Bayesian framework, it needs defining prior knowledge which allows to properly account for subsurface heterogeneity. The associated difficulty lies in estimating the probability density over a non-linear space of parameters and requires using a Monte Carlo method for generating many parameter fields and forward model runs. Depending on the physical problem, the forward model can be computationally expensive (especially true for transient groundwater flow or contaminant transport models); the inversion would typically require long runs and using high-performance computing resources.

Machine learning, including deep learning, has gained momentum in water research (Shen et al., 2018), and can be used to improve the efficiency of the inverse methods (Marçais and de Dreuzy, 2017). There are two main areas of application of machine learning related to the inverse problem: first, using machine learning techniques for modeling the prior knowledge; second, emulating the forward problem.

Concerning the generation of samples from the prior distribution, generative adversarial networks (GAN) are interesting because of their ability to generate geologically realistic models. Despite significant training times, they are attractive for the fast generation of fields and because they offer a low dimensional parametrization, which allows an efficient exploration of model space (Laloy et al., 2017; Chan and Elsheikh, 2020). As a consequence, GANs have been successfully used in Markov Chain Monte Carlo algorithms for solving groundwater inversion problems (Laloy et al., 2017, 2018). Due to the possibility of computing gradients in GAN models space, deterministic inversion using GANs is also possible but can be hindered by the non-linearities of the problem (Laloy et al., 2019). Simpler models, such as Support Vector Machines (SVM) were also used to construct informative geological prior to constrain sampling for realistic reservoir models (Arnold et al., 2019).

Concerning the emulation of the forward problem, machine learning can also be used. For example, Tripathy and Bilionis (2018) used deep neural network to construct a surrogate for a stochastic partial differential equations and employed it in the context of high dimensional uncertainty quantification. Laloy and Jacques (2019) tested machine learning algorithms (including deep neural nets, Gaussian processes, polynomial chaos expansion) to emulate reactive transport model and found that deep neural networks perform reasonably well in the context of quantifying uncertainty. Dagasan et al. (2020) showed how GAN can emulate steady-state flow solver and used it in the posterior population expansion algorithm (Jäggli et al., 2018), showing that the results of the inversion were of similar quality as those obtained using the numerical flow solver.

All these approaches rely on machine learning techniques built specifically for a problem at hand and replacing state-of-the-art methods for either modeling the prior, or emulating the forward problem.

In this paper, we propose a slightly different approach based on a machine learning classifier, and not substituting the prior geostatistical model, nor the forward solver. We use the posterior population expansion method (PoPEx) to invert a categorical field and our aim is to accelerate the convergence of the algorithm. During the Monte Carlo exploration of the model space, models are generated along with the forward operator results. These data are then used to train a classifier, which predicts if models would have high or low likelihood and whether they would contribute much to the posterior distribution. Then, the classifier can decide if a forward operator should be called (when the parameter field is predicted to be favorable) or if a model can be discarded, saving computational time. This approach is generic, as it does not rely on a specific geostatistical prior model, neither on a forward solver type. It is similar in spirit to the approach of building a surrogate or emulated model (like Laloy et al., 2019; Dagasan et al., 2020), but instead of reproducing the forward response, it predicts if the response would match well with the observed data. A similar idea was proposed by Demyanov et al. (2010), where SVM classifier was applied to separate high likelihood models while stochastically sampling parameters space for reservoir predictions.

To test the efficiency of the proposed method, We consider an alluvial aquifer. Its geological heterogeneity is modeled using multiple-point statistics. The inverse problem consists in interpreting one tracer test. Once the geological heterogeneity and its remaining uncertainty is identified, the resulting distribution of geological fields is used to predict the 10-days capture zone. This problem is used since it is a frequent question in applied hydrogeology. One has to interpret tracer test data to then delineate protections zones around future drinking water production wells. Therefore, in addition to the analysis of the efficiency of the inverse method, we check also the quality of this prediction as compared to the reference.

## 2. Methods

In this section, we present a brief review of the posterior population expansion algorithm, and the machine learning schemes with their performance metrics used in this study. Finally, we explain how the schemes are used to accelerate PoPEx algorithm.

### 2.1. Inverse Problem and PoPEx Algorithm

PoPEx has been previously introduced and presented in detail in Jäggli et al. (2017) and Jäggli et al. (2018). It solves the inverse problem in a probabilistic manner. First, we explain how the problem is framed; then, we review the algorithm and its parameters; finally, we show how the solution can be used to generate a prediction.

#### 2.1.1. Probabilistic Formulation

We will consider that $d_{obs}\in {\mathbb{R}}^{n}$ is a data set obtained from an experiment with *n* defining the number of data points. The data are physical state variables, for example: hydraulic heads, contaminant concentrations, or flow rates. Let $\text{g}:M\to {\mathbb{R}}^{n}$ be the forward operator, mapping from the model space M to the data space ℝ^*n*^. The model space defines the set of physical parameters, which fully describe the system (for example subsurface parameters) and allows to solve the forward problem (for example a groundwater flow problem). The forward operator generates the observable data given the model. In our case, the forward operator is a groundwater flow and transport model, and the model space is a set of all possible geological realizations of the subsurface, which map to permeability, porosity and specific storage.

The probabilistic solution of the inverse problem is given by (Tarantola, 2005):

$$\begin{array}{l} \sigma \left(\text{m}\right)=c\rho \left(\text{m}\right)L\left(\text{m}\right),\;\text{m}\;\in M \end{array}$$

with *σ* the posterior probability density, *ρ* the prior probability density, *L* the likelihood, and *c* a normalization constant. The prior probability density is defined by expert knowledge about the parameter field. For example, it can constrain the type of geology which is considered. The likelihood evaluates the mismatch between the data and simulated values (output of the forward operator).

#### 2.1.2. PoPEx Algorithm

In the following, we provide a rapid and brief overview of PoPEx. The posterior population expansion algorithm (Jäggli et al., 2018) is a modified adaptive importance sampler. It iteratively expands a sampled model set and learns the underlying probability distribution. It is designed for solving inverse problems when the space M contains categorical models. It requires a conditional geostatistical tool (e.g., a geostatistical algorithm which can generate a new model **m** from space M given conditioning points); and a forward solver which computes the likelihood given the model. At each iteration *k* it expands the sampled model set and uses all previously generated models and their likelihoods to define the conditioning set for the next model **m**_*k*+1_. The number of conditioning points at every iteration is uniformly drawn from {0, …, *n*_*c*_}, where *n*_*c*_ is the maximal number of conditioning points — a parameter specified by the user. Then, the new model is generated by the geostatistical tool honoring the imposed conditioning data and the forward solver computes its likelihood. The PoPEx algorithm stops after specified number of iterations *N* given by the user.

The choice of the conditioning data locations is guided by the Kullback-Leibler divergence (KLD) map computed between two discrete probability distributions *P* and *Q* on the probability space X and at each point in the domain:

(1) $$\begin{array}{r} D\left(P^{k}\|Q\right)=\underset{x\in X}{\sum }P^{k}\left(x\right)\log\left(\frac{P^{k}\left(x\right)}{Q\left(x\right)}\right). \end{array}$$

*Q* = {*q*_1_, …, *q*_*s*_} corresponds to the prior probability maps for each category, with *s* the number of categories (e.g., geological facies). $P^{k}=\left\{p_{1}^{k},\ldots ,p_{s}^{k}\right\}$ correspond to the maps of facies probabilities but weighted by the normalized likelihoods estimated at iteration *k*: $\tilde{L}\left(m_{j}\right)=L\left(m_{j}\right)/\left(\overset{k}{\underset{r=1}{\sum }}L\left(m_{r}\right)\right)$. The higher the KLD for a given point, the more likely it is chosen as a conditioning data location. Once conditioning point locations are determined, their values are sampled from the local *P* distribution.

#### 2.1.3. Prediction

Typically, solutions of the inverse problem are used to generate some predictions. Let *f* be the function mapping from models to predictions, and *μ* the expected value of some quantity of interest (e.g., prediction of hydraulic head, capture zone of a well):

(2) $$\mu =\int_{M}\sigma (m)f(m)dm.$$

PoPEx uses the set of generated models to approximate (2):

$$\begin{array}{l} \mu =\overset{N}{\underset{i=0}{\sum }}{\hat{w}}_{i}f\left({\text{m}}_{i}\right), \end{array}$$

with ${\hat{w}}_{i}$ a normalized *corrected* weight of the model *i*. The *corrected* weights are computed from the sampling weights *w*_*i*_:

$${\hat{w}}_{i}=\frac{w_{i}^{\alpha }}{\sum_{i=1}^{N}w_{i}^{\alpha }},$$

with *α* the correcting factor. The weights *w*_*i*_ are given by:

(3) $$\begin{array}{r} w_{i}=L\left({\text{m}}_{i}\right)\frac{\rho \left({\text{m}}_{i}\right)}{\phi_{i}\left({\text{m}}_{i}\right)}, \end{array}$$

where *ρ* is the prior distribution function and *ϕ*_*i*_ is the sampling distribution used at the iteration *i*. The weights need to be adjusted, as with large model spaces the distribution $W^{N}=\left\{w_{1},\ldots ,w_{N}\right\}$ can be dominated by few very large samples. The effective sample size *n*_*e*_ provides a measure of the skewness of the distribution:

(4) $$n_{e}(W^{N})=\frac{\sum_{i=1}^{N}w_{i}^{2}}{{\left(\sum_{i=1}^{N}w_{i}\right)}^{2}}$$

Correcting the weights consists of finding the factor *α* ∈ (0, 1] as close (or equal) to 1 such that the effective number of weights of the set {*w*_1_, …, *w*_*N*_} is at least *l*_0_, with *l*_0_ specified by the user.

### 2.2. Machine Learning Methods

Binary classification is a supervised learning task that has been extensively studied in the context of predictive data analytics (Kelleher et al., 2015), statistical learning (Hastie et al., 2009), and deep learning (Goodfellow et al., 2016). It consists in predicting the binary label (binary class, encoded as 0 or 1) given the input data (features). If *X* is the input (a feature vector, containing categorical or continuous variables), a binary classifier $\hat{f}$ maps *X* to the binary label *ŷ*: $\hat{f}\left(X\right)=ŷ$ with *ŷ* ∈ {0, 1}. The classifier must be fitted to known data with assigned labels, and then it can be used to generate predictions on previously unseen data. In this work, we considered three classifiers: AdaBoost, Random Forest, and Convolutional neural network (CNN). Here, we briefly introduce these methods and the cross-validation technique which was used to tune parameters of the classifiers and select the best algorithm. We refer to Hastie et al. (2009) for introductions about AdaBoost, Kelleher et al. (2015) about decision trees, Breiman (2001) about Random Forest, and Goodfellow et al. (2016) about CNN and deep learning in general.

#### 2.2.1. Classifiers

AdaBoost is a boosting method (Freund and Schapire, 1997) which produces a sequence of weak classifiers. The weak classifiers are iteratively fitted to the data with weights modified at each step. More weight is given to previously missclassified samples (initially weights are equal). The final prediction of the AdaBoost algorithm is a majority vote of all classifiers. Typically, the weak classifiers used in AdaBoost are shallow decision trees. The error on the training sample is the average of the fraction of misclassified samples. The fitting is finished when perfect fit is achieved or when the total number of estimators has been reached.

Random Forests (Breiman, 2001), similarly to AdaBoost, rely on ensembles of decision trees. The decision trees are typically deeper than in AdaBoost and they are trained on data randomly sampled from the input. The sampling distribution from which the training data is drawn is the same for all trees in the forest. The main parameters of the method are the tree depth and the number of trees that form the forest. Breiman (2001) claimed that random forests compare favorably to AdaBoost, yielding similar errors and being more robust with respect to noise.

For gridded inputs, convolutional neural networks (LeCun et al., 1989) proved to be effective. Convolutional neural networks are a special type of neural nets (Goodfellow et al., 2016), composed of convolutional layers, combined with activation, pooling and dense layers. They are especially suitable for object recognition and are state-of-the art classifiers for working with images.

#### 2.2.2. Cross-Validation

Cross-validation is a technique which allows to estimate the error (or a score) of the classifier on the unseen data. In K-fold cross-validation the whole training data is divided into *K* subsets and *K* iterations are performed. In each iteration *iter* = 1, …, *K*, the *iter* subset in a row is removed from the data to form the validation set. The rest of the data becomes the training set. The classifier is fitted to the training set and the error (or a score) is computed using the validation set: predictions are made on the validation set and they are compared to true values. Then, gathering the output of *K* iterations, one can compute the statistics of the error or the score function. Kohavi (1995) argued that the best choice is *K* = 5 or *K* = 10.

#### 2.2.3. Performance Measures

We introduce here the commonly used performance measures for binary classifiers, which are based on the confusion matrix. Let us suppose that the classification results in TP true positives, TN true negatives, FP false positives and FN false negatives. Precision is defined as follows:

(5) $$\begin{array}{r} \text{precision}=\frac{\text{TP}}{\left(\text{TP }+\text{ FP}\right)}, \end{array}$$

it describes how confident we can be that the predicted positive instance is correct. Recall is given by:

(6) $$\begin{array}{r} \text{recall}=\frac{\text{TP}}{\left(\text{TP }+\text{ FN}\right)}, \end{array}$$

and it describes the ability of the model to find all positive instances. The harmonic mean of precision and recall is F score (or *F*_1_ score) and it is generalized by *F*_*β*_ score (Rijsbergen, 1979):

(7) $$\begin{array}{r} F_{\beta }=\left(1+\beta^{2}\right)\frac{\text{precision }\cdot \text{ recall}}{\beta^{2}\text{precision }+\text{ recall}}, \end{array}$$

where *β* parameter describes how more recall is important over precision. For *β* = 1, the *F*_*β*_ falls back on standard F score which is a harmonic mean of precision and recall.

### 2.3. Accelerating PoPEx

At each PoPEx iteration *k*, a new model **m**_*k*_ is generated. Instead of feeding it directly to the forward solver, a learning scheme can predict if the model's likelihood is significant enough to contribute to the solution of the inverse problem. If the model is not especially useful, it can be discarded; its likelihood is set to 0. Otherwise, if the learning scheme predicts that the model is good enough, the forward solver is called and the exact likelihood of the model is computed. In this way, the expensive forward solver is not called for every model. Then, PoPEx proceeds to draw another model and the procedure is repeated. The discarded models do not contribute to the solution; even if the learning scheme makes a mistake, it will not bias the results. Discarding useful models or marking low-likelihood models as good, would only slow down the convergence of PoPEx but would not introduce incorrect likelihood estimations.

#### 2.3.1. Application of Learning Scheme

The learning scheme needs to be trained before being used with PoPEx. To this end, a sufficient dataset of pairs (**m**_*i*_, *L*(**m**_*i*_)) must be generated. To achieve this, we first run PoPEx in the normal mode, for a specified number *n*_*t*_ of iterations, evaluating all models using the forward solver.

Then, the machine learning scheme is trained using the pairs {(*X*_*i*_, *y*_*i*_), *i* = 1, …, *n*_*t*_} with *X*_*i*_ the input data and *y*_*i*_ the classification labels. *X*_*i*_ can be models **m**_*i*_ or some data obtained by transforming **m**_*i*_, for example collection indicator variables, or physical properties (porosity, conductivity) derived from **m**_*i*_. *y*_*i*_ ∈ {0, 1} is a binary label describing if a model is useful (0 meaning an insignificant model with low likelihood and 1 – important model with high likelihood). We propose to sort all available likelihoods: {*L*(**m**_*i*_), *i* = 1, …, *n*_*t*_}, define a ratio *r* ∈ (0, 0.5] and set *y*_*i*_ = 1 if *L*(**m**_*i*_) is among the *rn*_*t*_ highest likelihoods, and *y*_*i*_ = 0 otherwise.

Once the learning scheme has been trained, the classifier is used to classify each new model **m**_*j*_, *j* > *n*_*t*_. Let *ŷ*_*j*_ be the prediction of the true label *y*_*j*_. If *ŷ*_*j*_ = 1, the model **m**_*j*_ is fed to the forward solver and its true likelihood is evaluated. If ŷ_*j*_ = 0, we set *L*(**m**_*j*_) = 0 and the forward solver is not called. The true likelihood of the model remains unknown and the model does not contribute to the solution of the inverse problem, nor influences the sampling scheme of PoPEx.

This methodology is motivated by the fact that often only very few models have high likelihood (Jäggli et al., 2018). Correcting of the prediction weights was designed to deal with this problem. Therefore, it is justified to discard models with low likelihood, as they would not influence the PoPEx predictions anyway. Applying a perfect classifier in this way, should produce the same results as the original PoPEx algorithm.

A classifier yields false positives and false negatives. False positives (e.g., classifying low-likelihood models as useful) cause that an uninteresting model is being fed to the forward solver. A classifier producing a lot of false positives would have a weaker speed-up abilities. False negatives are more problematic, as it means that good models are discarded. A lot of false negatives would slow down the convergence of PoPEx, as the sampling scheme does not benefit from updating KLD maps and learns more slowly.

#### 2.3.2. Speed-Up Score and Evaluation Metrics

Given the training set for the classifier, it is useful to estimate the possible speed-up when applying it in the PoPEx scheme. We suppose here that we want to achieve an inversion result equivalent (in the sense of prediction error) to running plain (standard) PoPEx for *N* iterations. Let us compare the total cost of expanding an ensemble of *n*_*t*_ PoPEx models to *N* models and the total cost of expanding to a bigger ensemble with the machine learning scheme with the equivalent number of significant models. More models are needed to account for the fact that due to the learning scheme, some good models are discarded. To simplify the calculation, we suppose that generated new models have the same proportion of good and bad models and we neglect the fact that using ML scheme influences PoPEx sampling and the quality of generated models.

Let *c*_*m*_ be the computational cost of generating a model **m**, *c*_*g*_ the cost of running the forward problem solver and we set *c* = *c*_*m*_/*c*_*g*_. We assume that the forward solver is more expensive than running the geostatistical model, thus *c* < 1. The total computational cost of expanding the ensemble, e.g. adding *N*_*t*_ = *N* − *n*_*t*_ models without ML is *N*_*t*_ · (*c*_*m*_ + *c*_*g*_). Suppose that out of *N*_*t*_ models, ML scheme generates TP true positives ($\hat{y_{i}}=1$ and *y*_*i*_ = 1), TN true negatives ($\hat{y_{i}}=0$ and *y*_*i*_ = 0), FP false positives ($\hat{y_{i}}=1$ and *y*_*i*_ = 0) and FN false negatives ($\hat{y_{i}}=0$ and *y*_*i*_ = 1). More models need to be generated to account for the fact that some positives are not detected, so the total number of models *N*_*t*_ must be multiplied by (TP + FN)/TP. All true and false positives require the forward solver, thus the total cost with ML will be:

$$\begin{array}{l} \frac{\text{TP }+\text{ FN}}{\text{TP}}\left(c_{m}N_{t}+c_{g}\left(\text{TP}+\text{FP}\right)\right). \end{array}$$

We propose to use the ratio of the total computational cost without ML divided by the cost when using ML scheme as a speed-up estimator. Let us call it *s-score* (s_score_):

$${\text{s}}_{\text{score}}=\frac{N_{t}\cdot (c_{m}+c_{g})}{\left[c_{m}N_{t}+c_{g}(\text{TP}+\text{FP})\right]\;\cdot \;\left(\text{TP}+\text{FN}\right)/\text{TP}}$$

Using the definitions for precision and recall (5, 6) and the ratio of “good” models TP + FN = *rN*_*t*_, we obtain:

(8) $$\begin{array}{r} {\text{s}}_{\text{score}}=\frac{N_{t}\left(c_{m}\;+\;c_{g}\right)}{\frac{1}{\text{recall}}c_{m}N_{t}\;+\;c_{g}\frac{1}{\text{precision}}rN_{t}}=\frac{1\;+\;c}{\frac{c}{\text{recall}}\;+\;\frac{r}{\text{precision}}}. \end{array}$$

When generating a model is significantly cheaper than running the forward solver, *c* → 0 and we obtain a convenient approximation:

(9) $$\begin{array}{r} {\text{s}}_{\text{score}}\approx \text{precision}/r, \end{array}$$

useful to evaluate rapidly if a learning scheme is potentially a good candidate for accelerating PoPEx sampling. If *c* values are greater than 1, the machine learning scheme is not interesting to apply, even if the recall is high. Smaller *r* would yield potentially large *s-score* if the precision is high. The *s-score* can also be expressed in terms of *F*_*β*_ score (7):

$$\begin{array}{l} {\text{s}}_{\text{score}}=\frac{1\;+\;c}{r\;+\;c}F_{\beta }, \end{array}$$

where *β*^2^ = *c*/*r*. In this case *β* is lower than 1 and close to 0, which attributes more importance to precision than recall.

## 3. Test Case

We consider a 2D synthetic reference field representing a fluvial aquifer. The data used for the inversion is the synthetic tracer breakthrough curve recorded at the pumping well. The reference probabilistic solution for the inverse problem is obtained after 40,000 PoPEx iterations, it will be used to assess the solutions generated using the learning schemes.

### 3.1. Synthetic Model of a Fluvial Aquifer

**Figure 1:**
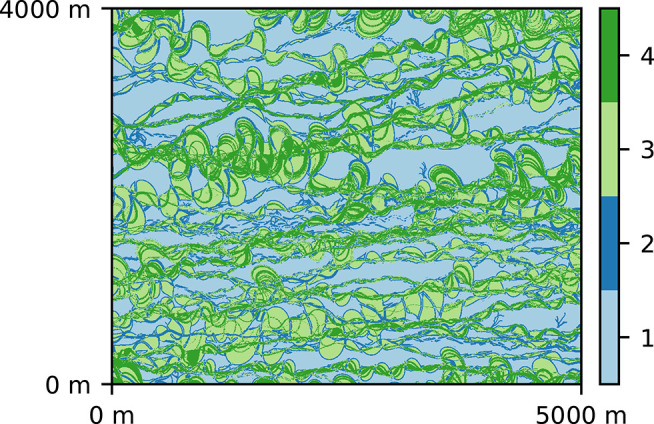
Training image used for generating the synthetic reality and in the inversion process.

**Figure 2:**
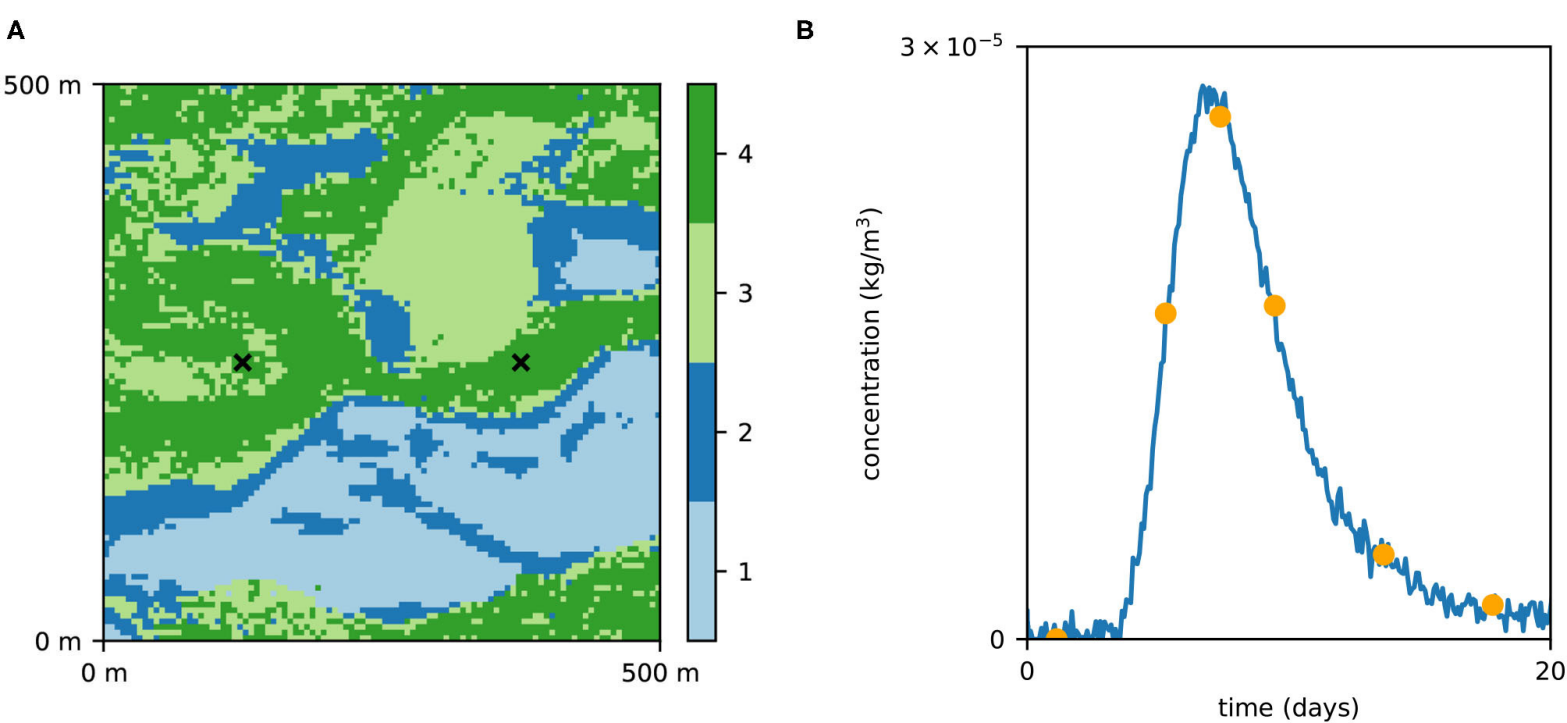
The reference field **(A)** used as the synthetic reality (considered unknown) and the corresponding concentration curve **(B)**. The orange dots correspond to data which were used for likelihood calculation.

The 2D geology is modeled by multiple-point statistics (MPS), which allows to account for subsurface heterogeneity and represent categorical fields (Mariethoz and Caers, 2015). The MPS algorithm requires a training image (TI), an example of the field which is used as a spatial pattern database. Here, we consider a TI composed of 4 geological facies, representing a fluvial aquifer (Figure 1), and first published by Jäggli et al. (2018). The TI has a dimension of 1,000 pixels by 800 pixels with cell size of 5 m by 5 m. To obtain the synthetic reference field (Figure 2A), we performed a Direct Sampling (Mariethoz et al., 2010) simulation on a regular grid representing 500 m by 500 m area (100 × 100 pixels). The DeeSse code with multi-resolution capabilities (Straubhaar et al., 2020) was used with the following parameters: 2 pyramid levels (with a pyramid for each indicator variable) with reduction factor 2 at each level and each direction (*x, y*); search neighborhood radius: 40 in each direction, number of neighboring nodes: 60, distance threshold: 0.01, maximal scan fraction: 0.04. The reference realization was generated with seed value of 201,913. Moreover, points at *x* = 374.5 m, *y* = 249.5 m and *x* = 124.5 m, *y* = 249.5 m are considered to have known facies of category 4, in other words conditioning data is imposed in these two locations with value 4.

### 3.2. Groundwater Flow and Transport

The aquifer is a confined fluvial aquifer with thickness of 10 m and modeled as a single layer. Each geological facies has a uniquely defined physical properties (hydraulic conductivity, porosity and specific storage) and can be interpreted as different rock type: silt, fine sand, coarse sand, and gravel (Table 1). The maps of conductivity, porosity and specific storage on a 100 × 100 regular grid obtained by means of DeeSse simulations are refined by factor of 5 in each direction, resulting in a 500 × 500 m model with cell size 1 m by 1 m. The value of a parameter at each location is equal to its value in the parent cell.

**Table 1:** Physical properties associated with different geological facies.

**Facies ordinal**	**1**	**2**	**3**	**4**
Facies	Silt	Fine sand	Coarse sand	Gravel
Conductivity (m/s)	10^−5^	10^−4^	10^−3^	10^−1^
Porosity	0.40	0.35	0.30	0.25
Specific storage (m^−1^)	10^−3^	5 × 10^−4^	10^−4^	10^−5^

A pumping well is placed at *x* = 374.5 m, *y* = 249.5 m and it pumps water with constant rate of 0.07 m^3^/s. The left (west) boundary is at constant head 0.5 m, the right boundary (east) at constant head 0 m and the hydraulic heads at top (north) and bottom (south) boundaries are linearly interpolated between 0.5 m and 0 m. The groundwater flow and transport model was implemented using the flopy python package (Bakker et al., 2016). The steady-state solution of the flow problem is used as initial condition for a transient groundwater flow and transport simulation of a tracer test. The injection well is placed at *x* = 124.5 m, *y* = 249.5 m. The tracer is injected at a constant concentration of 1 kg/m^3^ with a constant injection rate of 1 m^3^/h, so that a total of 1 m^3^ water is injected during 1 h.

The total simulation time is 1 h plus 20 days, discretized in 36 time-steps during initial 1 h and 240 time-steps during the rest of the simulation time. During this period, the flow is modeled in transient regime to account for the perturbation due to the injection. The transport simulation is done with the following parameters: diffusion coefficient of 1 × 10^−9^ m^2^/s; longitudinal dispersivity of 4 m and transversal dispersivity of 0.4 m. The concentration curve at the pumping well was recorded and random Gaussian noise with mean 0 and standard deviation *σ*_*L*_ = 0.05 × 10^−5^ kg/m^3^ was added to emulate measurement error (Figure 2B).

### 3.3. Reference Solution of the Inverse Problem

**Figure 3:**
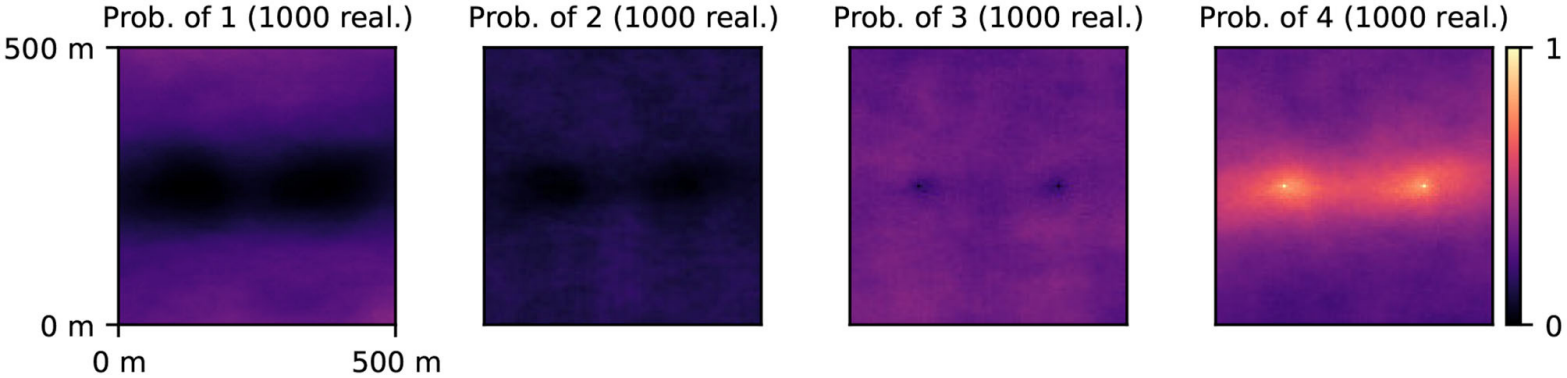
Prior probability maps associated with different geological facies (labeled 1, 2, 3, 4).

In this scenario, the prior distribution of the geological fields is modeled using the MPS technique with the same parameters as those used to generate the reference realization. Two conditioning points are imposed: the facies 4 (permeable, gravel) at the pumping well and injection well. A prior ensemble of 1,000 realizations was generated and facies probability maps (Figure 3) were used as input for the PoPEx algorithm.

The likelihood for this problem should be written as proportional to $\exp\left(-1/2\sigma_{L}\overset{n}{\underset{i=1}{\sum }}{\left({\text{g}}_{i}\left(\text{m}\right)-{\text{d}}_{i}^{obs}\right)}^{2}\right)$ with *σ*_*L*_ the standard deviation of measurement error, **g**(**m**) the simulated values of concentration of the model **m**, and **d**^*obs*^ the vector of measurements. However, when using this complete formula, the likelihood values are zero for most realizations (considering the fact that numerical representation of floating point numbers has a finite number of digits) and PoPEx converges very slowly (Jäggli et al., 2018). Therefore, to avoid this numerical issue, and following Jäggli et al. (2018), we considered a reduced data set including only six time steps to estimate the likelihood. The “sampled” likelihood formula was: $\exp\left(-1/2\sigma_{L}\underset{i\in I}{\sum }{\left({\text{g}}_{i}\left(\text{m}\right)-{\text{d}}_{i}^{obs}\right)}^{2}\right)$ with *I* = {50, 100, 125, 150, 200, 250}.

**Figure 4:**
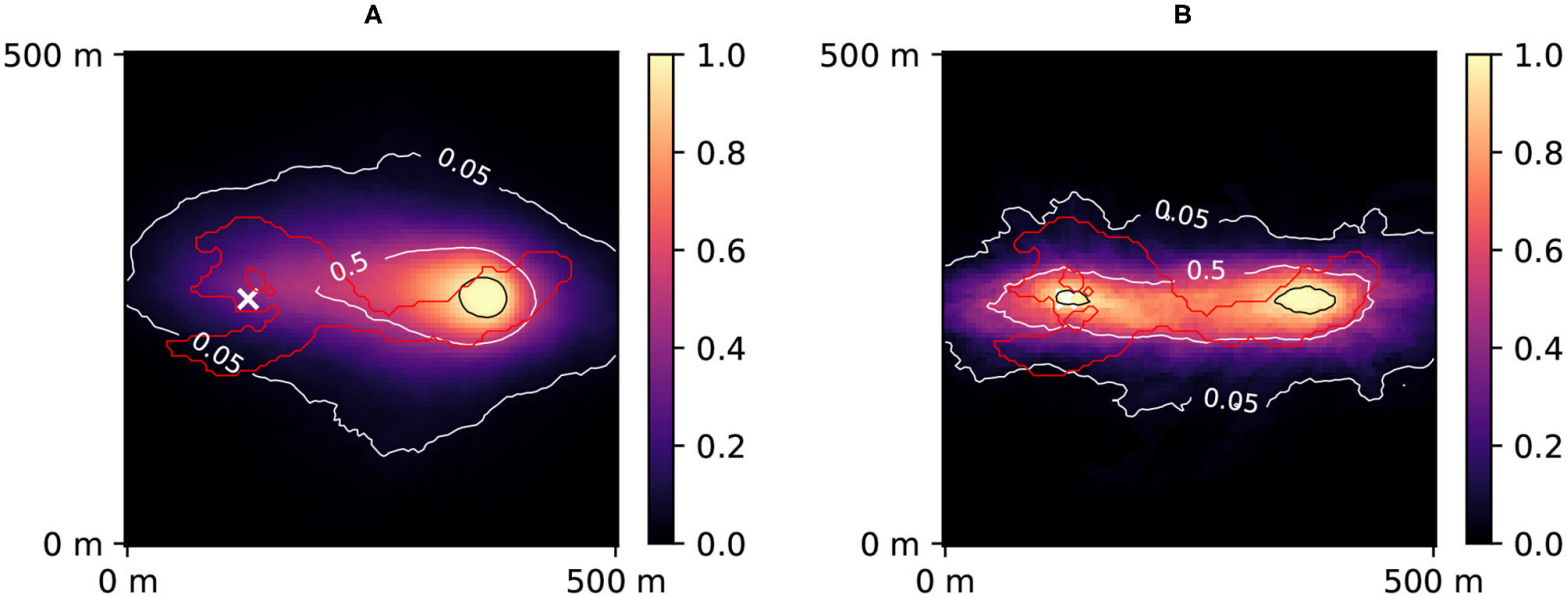
Prior **(A)** and posterior **(B)** probability maps of 10 days zone. The red contour corresponds to the reference 10 days zone (simulated using the synthetic reality; considered unknown). The white cross represents the injection well. The black contour corresponds to 95 % confidence. The posterior was obtained after 40,000 PoPEx iterations.

**Figure 5:**
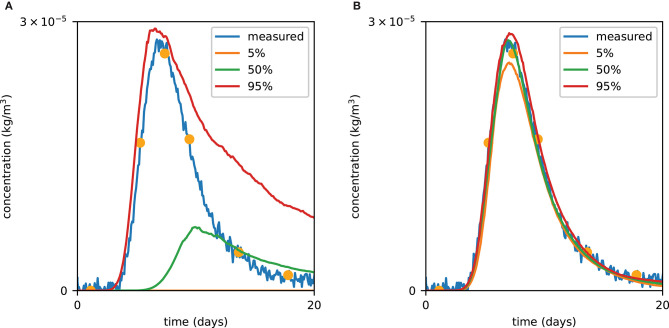
Prior **(A)** and posterior **(B)** tracer concentration curves. The orange dots correspond to data which were used for likelihood calculation. The posterior was obtained after 40,000 PoPEx iterations.

PoPEx was run for 40,000 iterations with *l*_0_ = 100 and *n*_*c*_ = 10. The 10 days groundwater capture zone (van Leeuwen et al., 1998) was predicted with the forward particle tracking module of flopy. The particle tracking was performed on the steady-state solution of the groundwater flow using the original grid composed of 100 × 100 pixels with the cell size of 5 m × 5 m. For each pixel in the domain, a probability of being in the 10 days zone was computed a priori (Figure 4A) and a posteriori (Figure 4B). Figure 5 shows the prior and posterior concentration probabilities at the pumping well.

### 3.4. Application Scenario

While it is possible to apply a classifier directly on a set of models **m**, in the context of groundwater transport it is favorable to transform the input prior to feeding it to the ML scheme. Below we describe how ML inputs are formed and specify the algorithm parameters.

#### 3.4.1. Transforming Model Into ML Input

The groundwater velocity vector (*v*_*x*_, *v*_*y*_) controls advection and hydrodynamic dispersion

(10) $$\begin{array}{r} v_{x}=-\frac{K}{n_{e}}\frac{\partial h}{\partial x},\;v_{y}=-\frac{K}{n_{e}}\frac{\partial h}{\partial y} \end{array}$$

with *K* the hydraulic conductivity, *n*_*e*_ the effective porosity and *h* the hydraulic head. The hydraulic heads are computed using a steady-state MODFLOW (flopy) simulation. Values of conductivity and porosity are defined according to Table 1. Two-point numerical derivative is then used to calculate the hydraulic gradient.

Given the model **m**_*i*_ which is 100 × 100 pixels, *X*_*i*_ is formed by stacking *v*_*x*_ and *v*_*y*_ (each of size 98 × 98 pixels) and normalizing them to the interval [0,1], using one global minimum and maximum (the maximal/minimal value found in all vectors *X*_*i*_, *i* = 1, …, *n*_*t*_). A 98 × 98 image with two channels is formed. The total number of features in the input is 98 × 98 × 2. For AdaBoost and Random Forest the input is flattened, while CNN benefits from the spatial arrangement of the features. The classification labels are obtained by labeling ratio *r* of best likelihoods as good (1), and the rest not significant (0), as described in section 2.3.1.

#### 3.4.2. Initial PoPEx Ensemble

**Figure 6:**
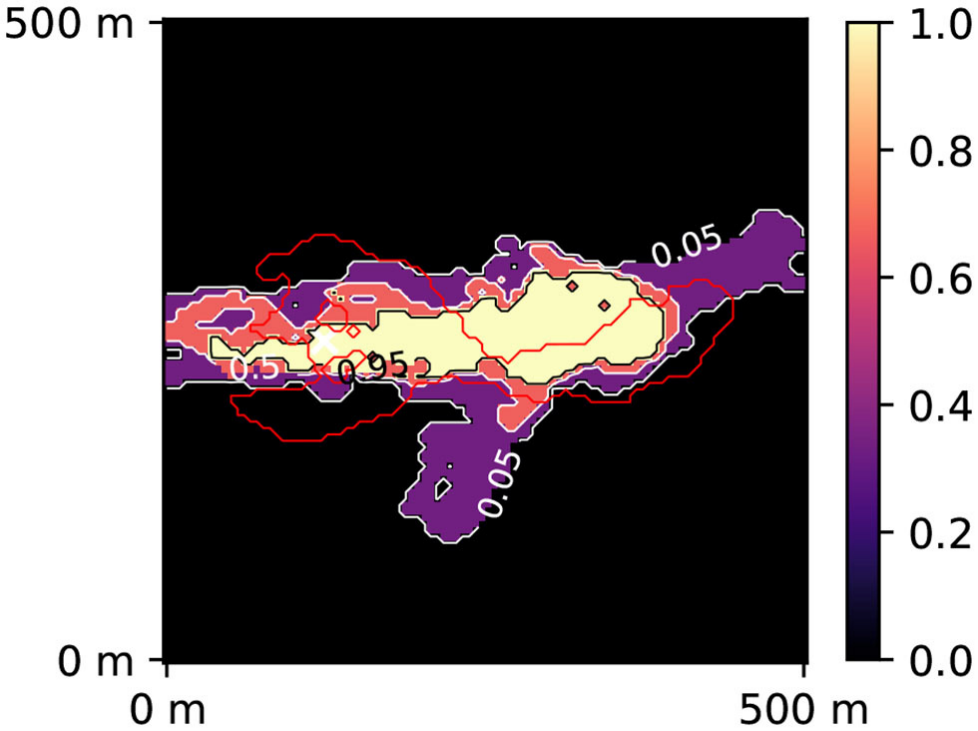
Posterior probability map of 10 days zone obtained after 500 PoPEx iterations. The red contour corresponds to the reference 10 days zone (simulated using the synthetic reality; considered unknown). The white cross represents the injection well.

We generated a PoPEx solution using only 500 iterations (Figure 6). The initial ensemble is needed for training the machine learning scheme. The generated set of models with their likelihoods will be used to tune parameters of the machine learning schemes, and to select the best classifier for two different ratios *r* = 0.4 and *r* = 0.2. To this end, we will apply five-fold cross-validation. For each ratio, parameters of all methods will be re-tuned and the corresponding best classifiers will be used for validating the ML-accelerated PoPEx.

### 3.5. Test Procedure

To test the methodology, we will compare the results obtained by the standard PoPEx algorithm after *N* = 2,000 iterations with the results obtained by ML-accelerated PoPEx algorithm after *N* = 4,000 iterations, taking into account computing time and error with respect to the reference solution. We will consider two cases: ratio *r* = 0.2 and *r* = 0.4. This test procedure will be repeated 4 times. Each time with a different initial PoPEx ensemble (neither being the ensemble used for ML parameter tuning).

To evaluate the results, the computing time and discrepancy with the reference solution (40,000 PoPEx iterations) will be computed. The discrepancy will be measured on the prediction: 10-days zone probability maps compared by means of then Jensen-Shannon divergence:

(11) $$\begin{array}{r} J\left(\hat{\mu }\|\mu^{ex}\right)=\frac{1}{2}\left(D\left(\hat{\mu }\|\bar{\mu }\right)+D\left(\mu^{ex}\|\bar{\mu }\right)\right) \end{array}$$

with $\hat{\mu }$ the estimated probability map, *μ*^*ex*^ the exact probability map, $\bar{\mu }=\left(\hat{\mu }+\mu^{ex}\right)/2$, and *D*(·‖·) Kullback-Leibler divergence (1).

## 4. Results

In the first test, we perform a comparison of the performances of three machine learning algorithms: AdaBoost, Random Forest, and CNN, and different ratios of models labeled as good (significant) models. This test corresponds to the practical application of the methodology to accelerate PoPEx: the choice of the ML method would be based on limited number of realizations without knowing the true solution. In the second test, we verify how the best performing methods according to the first step are able to accelerate the inversion.

Each inversion task was performed on a computing node composed of 2 Intel(R) Xeon(R) CPU D-1541 @ 2.10GHz CPUs running 64 threads in total. The total RAM of a node is 256 GB. PoPEx was run with 63 processes (workers) and one master process. Running inversion with *N* = 500 iterations required about 24 h to finish. The approximate average time required for completing one geostatistical simulation by the computing node is *c*_*m*_ = 6.3 s and for the forward solver: *c*_*g*_ = 1.7 × 10^2^ s; *c* = *c*_*m*_/*c*_*g*_ is approximately equal to 0.037.

### 4.1. Hyperparameter Tuning

The hyperparameter tuning was performed with the initial ensemble containing 500 realizations. Depending on the ratio, different number of models were marked as good: 100 models for *r* = 0.2, and 200 models for *r* = 0.4. The validation scores from five-fold cross-validation on these datasets were used to compare the models. The cases of two ratios are treated separately.

#### 4.1.1. Random Forest and AdaBoost

**Figure 7:**
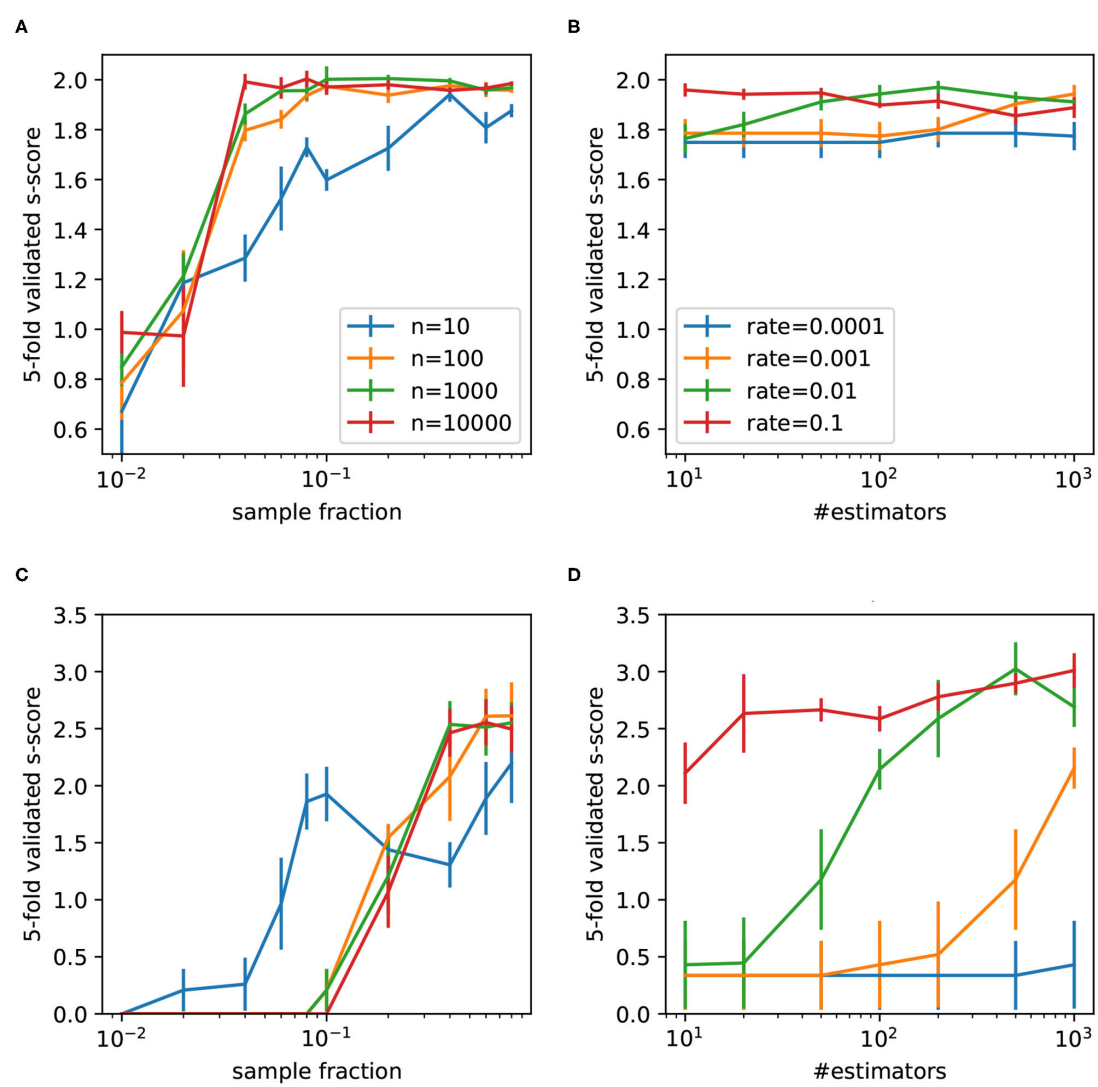
Five-fold cross-validated *s-scores* obtained by Random Forest **(A,C)** and AdaBoost **(B,D)** for two ratios 0.4 (upper row) and 0.2 (lower row). Mean values are reported with the standard error of the mean.

The Random Forest and AdaBoost were implemented using scikit-learn library (Pedregosa et al., 2011). The mean five-fold cross-validation *s-score* on the validation sets was used as performance metric. The Random Forest was tested with the following range of base estimators: 10 to 10,000. The number of samples (measured as fraction of all samples in the training dataset) were varied and scores reported for the ratio *r* = 0.4 (Figure 7A) and *r* = 0.2 (Figure 7C). The AdaBoost classifier was tested with the learning rates in the range 0.0001 to 0.1 and number of estimators varied between 10 to 1,000. The scores for the ratios *r* = 0.4 and *r* = 0.2 are shown in Figures 7B,D, respectively. In the case *r* = 0.4, Random Forest slightly outperformed AdaBoost. The following parameters were selected: number of classifiers 1,000 and fraction of samples 0.2. In the case *r* = 0.2 AdaBoost outperformed Random Forest. The corresponding optimal parameters are: number of estimators 500 and learning rate 0.01.

#### 4.1.2. CNN Architecture Selection

**Figure 8:**
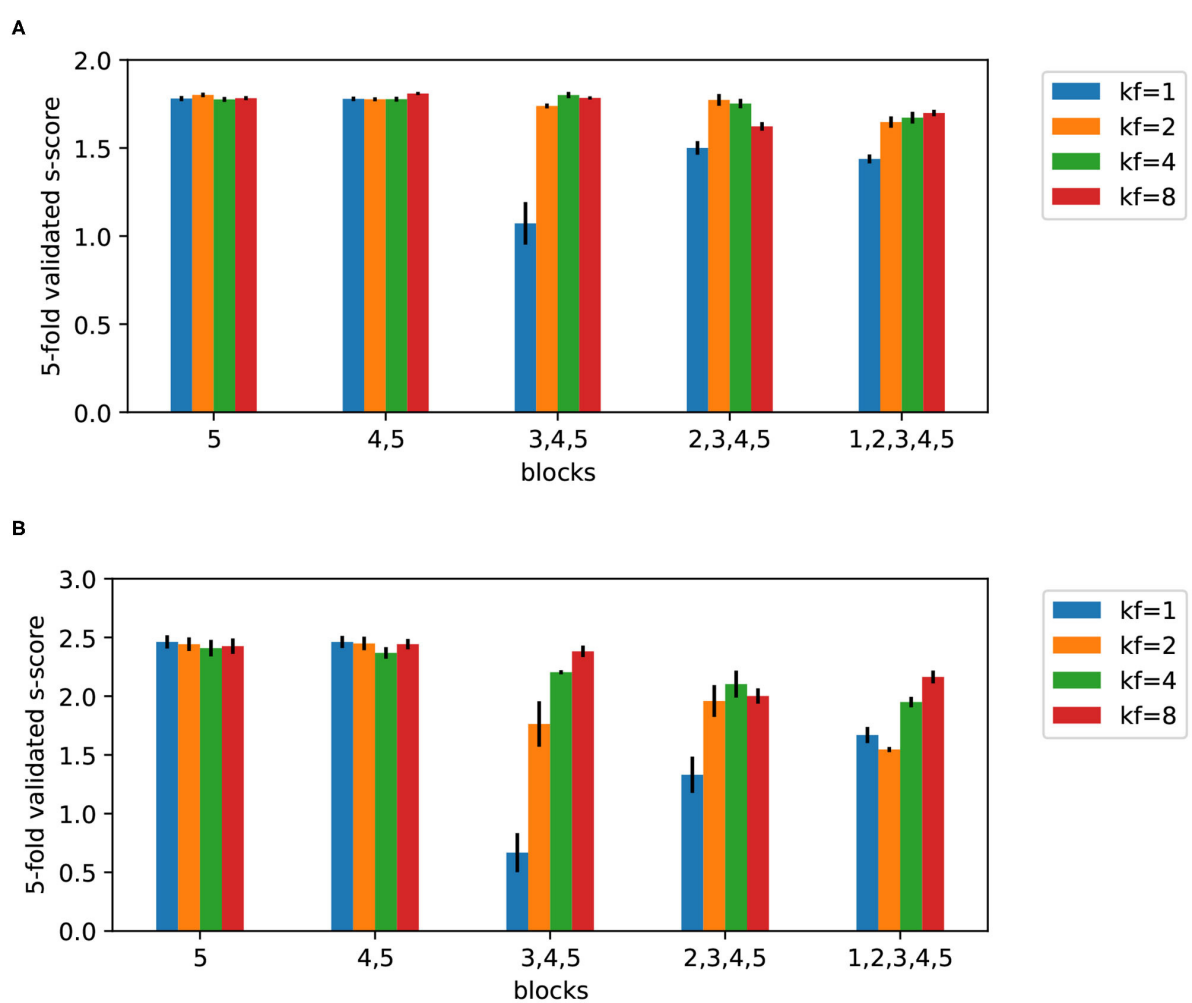
Five-fold cross-validated *s-scores* on test sets obtained by CNN for two ratios 0.4 **(A)** and 0.2 **(B)**, and different kernel factors (kf). Mean values are reported with the standard error of the mean.

The CNN was implemented using Keras library and the initial architecture (Table 2) is an adapted version of the AlexNet (Krizhevsky et al., 2012) to match the input size in our study. We considered the following variants of the initial architecture: included different blocks (1,2,3,4,5) and *kernel factors* (1,2,4,8). Five architecture variants were considered: first, including only block 5; second, including blocks 4,5; third, including blocks 3, 4, 5; etc. As block 4 and 5 are dense layers, the architecture with one and two blocks correspond to feed-forward neural nets. The remaining architectures are CNNs with two dense layers before the output layer. We also introduced a second architectural parameter, *kernel factor*. It was used to divide the kernel size of convolutional layers and number of nodes in the dense layers. A higher number decreases the number of parameters of the neural net. In other words, the *kernel factor* defines a number by which the last column in the Table 2 is divided. In this way, 20 variants of CNN architecture were formed. For each variant, a five-fold cross-validated learning curve was obtained and the epoch corresponding to the minimal validation loss was noted. The batch size was that of the full training set, and Adam optimizer was used with binary-crossentropy loss and constant learning rate 0.001. The loss is weighted according to class weights: *r* for the class 0 (not significant models) and 1 − *r* for the significant (“good”) models. The weights (as in the case of Random Forest) are used to correct the fact that the training set is not class-balanced; it forces the algorithm to pay more attention to the underrepresented class. Then, the five-fold cross-validated *s-score* after the optimal epoch was retained. This procedure was applied for ratio *r* = 0.4 and *r* = 0.2 (Figures 8A,B) respectively. Simpler architectures perform generally better than those containing all the blocks and simple feed-forward nets outperform the more complex architectures.

**Table 2:** Layers of the convolutional neural network used for binary classification.

**Block**	**Layer type, activation**	**Kernel/pool size**	**Output shape**	**#kernels or #nodes**
Block 1	Conv. 2D, ReLU	5 × 5, stride 2 × 2	(47, 47, 24)	24
	Max-pool	3 × 3, stride 2 × 2	(23, 23, 24)	
Block 2	Conv 2D, ReLU	5 × 5, stride 2 × 2	(19, 19, 64)	64
	Max-pool	3 × 3, stride 2 × 2	(9, 9, 64)	
Block 3	Conv 2D, ReLU	5 × 5, stride 2 × 2	(7, 7, 96)	96
	Conv 2D, ReLU	5 × 5, stride 2 × 2	(5, 5, 96)	96
	Conv 2D, ReLU	5 × 5, stride 2 × 2	(3, 3, 64)	64
	Max-pool	3 × 3	(1, 1, 64)	
Block 4	Flatten			
	Dense, ReLU		1,024	1,024
Block 5	Flatten			
	Dense, ReLU		1,024	1,024
Output	Dense, sigmoid		1	1

### 4.2. Choosing ML Algorithm

The five-fold cross-validated scores of tuned AdaBoost, Random Forest, and CNN algorithms are reported in Table 3. The following mean scores and standard errors of the mean are compared: *s-score* (according to the formula 8), precision, recall, and approximate *s-score* (precision/*r*). The *s-score* value, as explained in the methodology, can be interpreted as an optimistic estimator for the real speed-up value.

**Table 3:** Five-fold cross-validated performance of classifiers on the initial ensemble containing 500 realizations.

**Classifier**	**r**	***s-score***	**Precision**	**Appr. *s-score***	**Recall**
AdaBoost	0.4	1.98 ± 0.03	0.84 ± 0.02	2.10 ± 0.03	0.78 ± 0.05
Random Forest	0.4	2.01 ± 0.02	0.86 ± 0.02	2.15 ± 0.03	0.77 ± 0.03
CNN	0.4	1.82 ± 0.01	0.76 ± 0.01	1.91 ± 0.03	0.80 ± 0.03
AdaBoost	0.2	3.1 ± 0.3	0.77 ± 0.06	3.9 ± 0.3	0.48 ± 0.06
Random Forest	0.2	2.6 ± 0.2	0.95 ± 0.05	4.8 ± 0.3	0.21 ± 0.04
CNN	0.2	2.48 ± 0.06	0.56 ± 0.03	2.8 ± 0.2	0.63 ± 0.05

Table 3 shows that the *s-score* and the precision obtained with Random Forest and AdaBoost are higher than those obtained with CNN for both values of *r*. The recall values are similar for all classifiers for *r* = 0.4, and the recall for *r* = 0.2 is better for CNN than for AdaBoost and Random Forest. It means that AdaBoost and Random Forest were able to achieve a high proportion of good models among all labeled as good, but missed some good models. Consequently, these results confirm that the approximate *s-score* overestimated the actual ones.

### 4.3. Test of ML-Accelerated PoPEx

Random Forest with 1,000 estimators and samples fraction 0.4 was chosen for validating ML-accelerated PoPEx for ratio of 0.4, and AdaBoost with 500 estimators and learning rate 0.001 for validating ML-accelerated PoPEx for ratio of 0.2. In the validation experiment, PoPEx in the standard mode was run for *N* = 2,000 iterations and two ML-accelerated PoPEx versions were run for *N* = 4,000 iterations: RF-accelerated PoPEx with *r* = 0.4 and AdaBoost-accelerated PoPEx with *r* = 0.2.

For each of these three PoPEx modes, 4 independent runs were performed and averages used to compute the mean errors with respect to the reference solution. The total running times, and the number of models fed to the forward solver were recorded. The accelerated modes of PoPEx were trained once after *n*_*t*_ = 500 iterations and from that iteration, the ML schemes were used.

**Figure 9:**
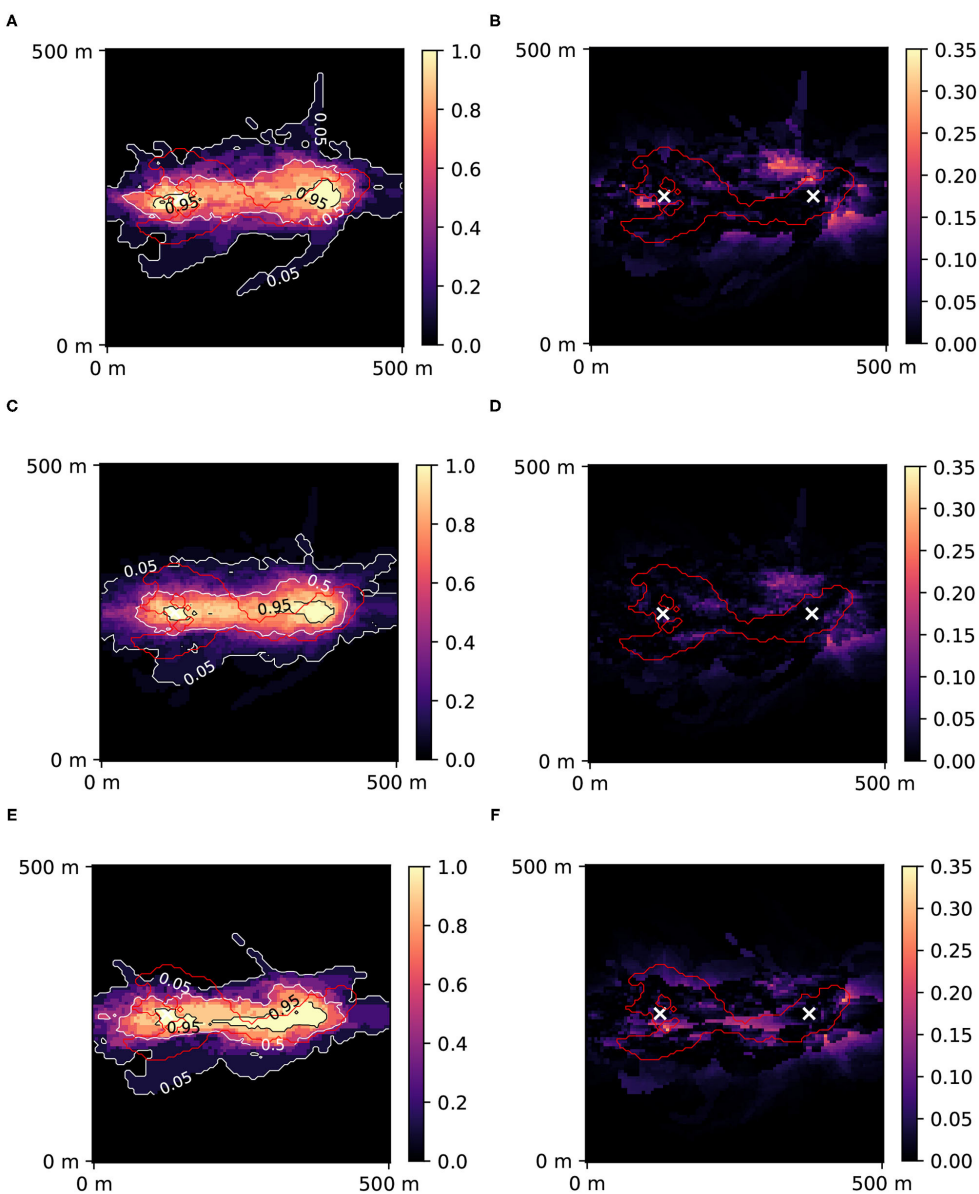
Posterior probability maps of 10 days zone with Jensen-Shannon error maps for standard PoPEx solution with *N* = 2,000 **(A,B)**, for RF-accelerated PoPEx with *N* = 4,000 and *r* = 0.4 **(C,D)**, and for AdaBoost-accelerated PoPEx with *N* = 4,000 and *r* = 0.2 **(E,F)**. The red contour corresponds to the reference 10 days zone. White crosses indicate positions of the injection well (left) and the pumping well (right).

Example results (1 out of 4) are shown in Figure 9, where predictions of 10-days zones after all iterations (2,000 for the standard mode, 4,000 for ML-accelerated) are reported with Jensen-Shannon error maps. The reference solution obtained after 40,000 iterations (Figure 4) was used in the Jensen-Shannon formula (11). In this example, the exact solution took 94 h 45 min to compute, the RF-accelerated with *r* = 0.4 took 88 h 12 min, and the AdaBoost-accelerated with *r* = 0.2 only 47 h 40 min. The lowest mean JS error achieved RF-accelerated mode with *r* = 0.4: 0.010, the exact mode: 0.015 and AdaBoost-accelerated mode with *r* = 0.2: 0.014.

**Figure 10:**
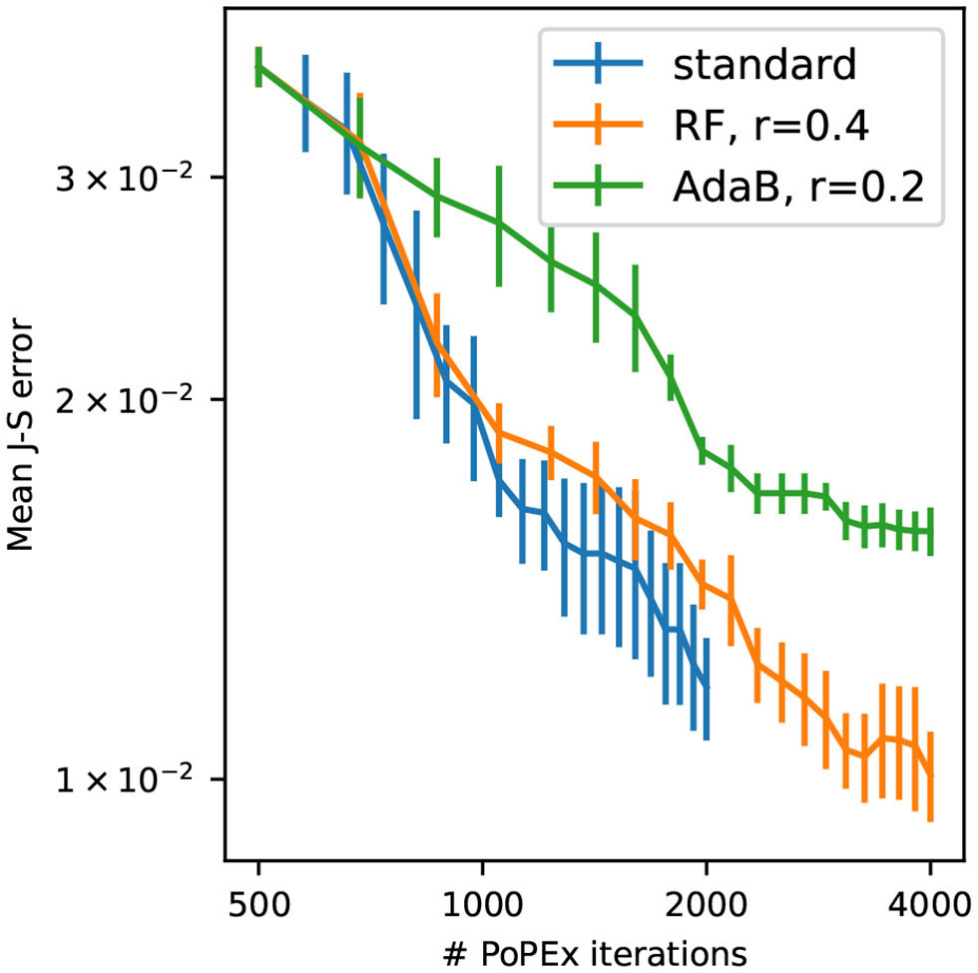
Convergence of standard PoPEx method compared with RF-accelerated PoPEx with ratio *r* = 0.4 and AdaBoost-accelerated with *r* = 0.2. Each point is an average over 4 PoPEx runs, and bars represent the standard error of the mean.

**Figure 11:**
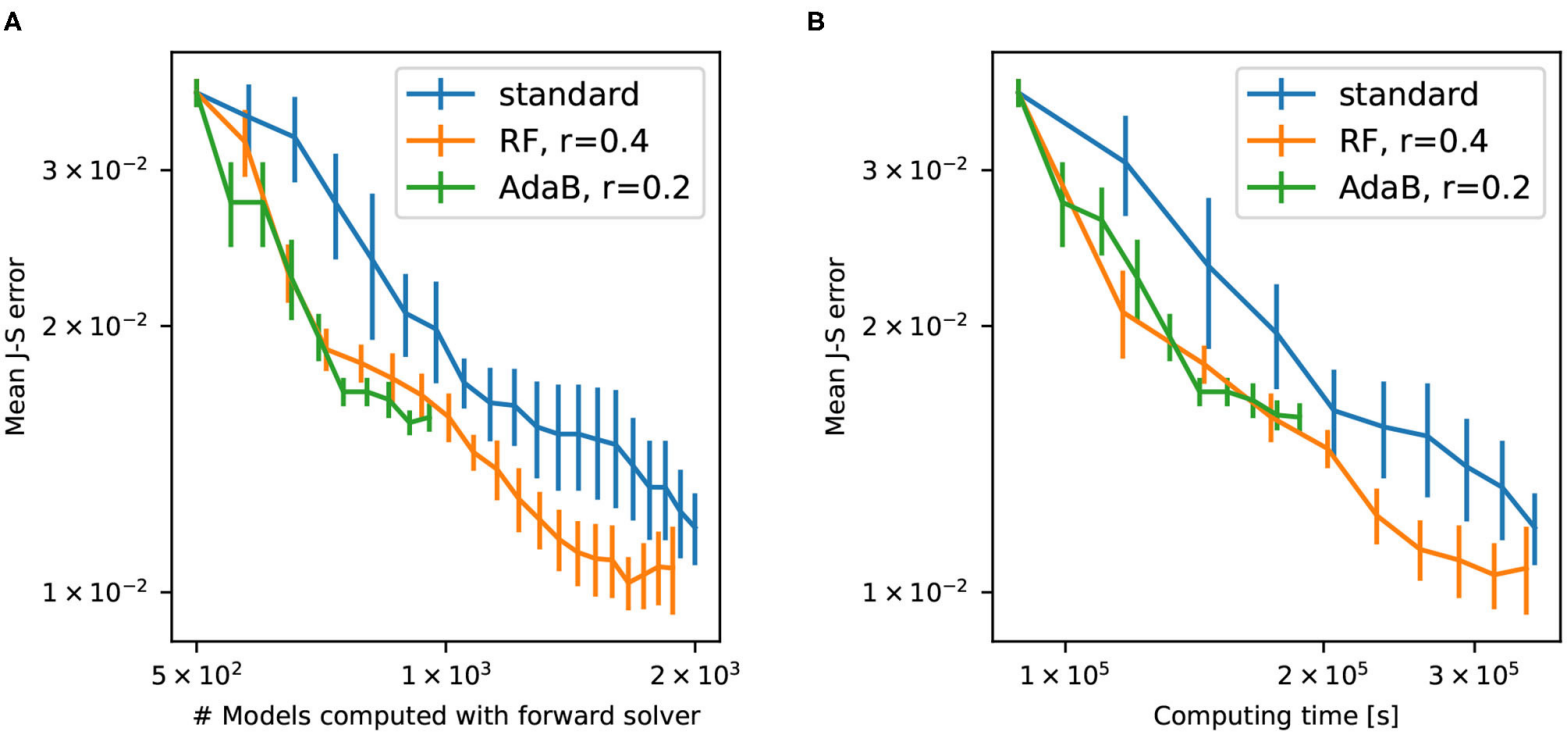
Error with respect to the number of models computed by the forward solver **(A)** and with respect to the computing time **(B)** for standard PoPEx, RF-accelerated PoPEx with *r* = 0.4, and AdaBoost-accelerated with *r* = 0.2. Each point is an average over 4 PoPEx runs, and bars represent the standard error of the mean.

Figure 10 shows the convergence of the different PoPEx modes with respect to the number of iterations. As expected, ML schemes slow down the convergence due to incorrect model classifications. Nevertheless, the convergence rate of the RF-accelerated mode with *r* = 0.4 is close to the standard mode. We can also see that this accelerated mode achieves the lowest values of mean error. Not all the generated models in ML-accelerated modes are sent to the forward solver, therefore the time required to achieve a specified number of iterations vary. This is why it is more representative of computing times to plot convergence with respect to the total number of models fed to the forward solver (Figure 11A) or to the total computing time (Figure 11B). These plots show that both ML-accelerated methods need computing fewer forward models to achieve the same error as the standard PoPEx version (Figure 11). These graphs show also that the convergence rate is similar for both values of *r*. If converted to total computing time, however, the RF-accelerated mode with *r* = 0.2 presents similar convergence rate as the standard mode, because many models need to be generated by the geostatistical method but are rejected. RF-accelerated mode with *r* = 0.4 still requires systematically less computing time than the standard mode.

**Figure 12:**
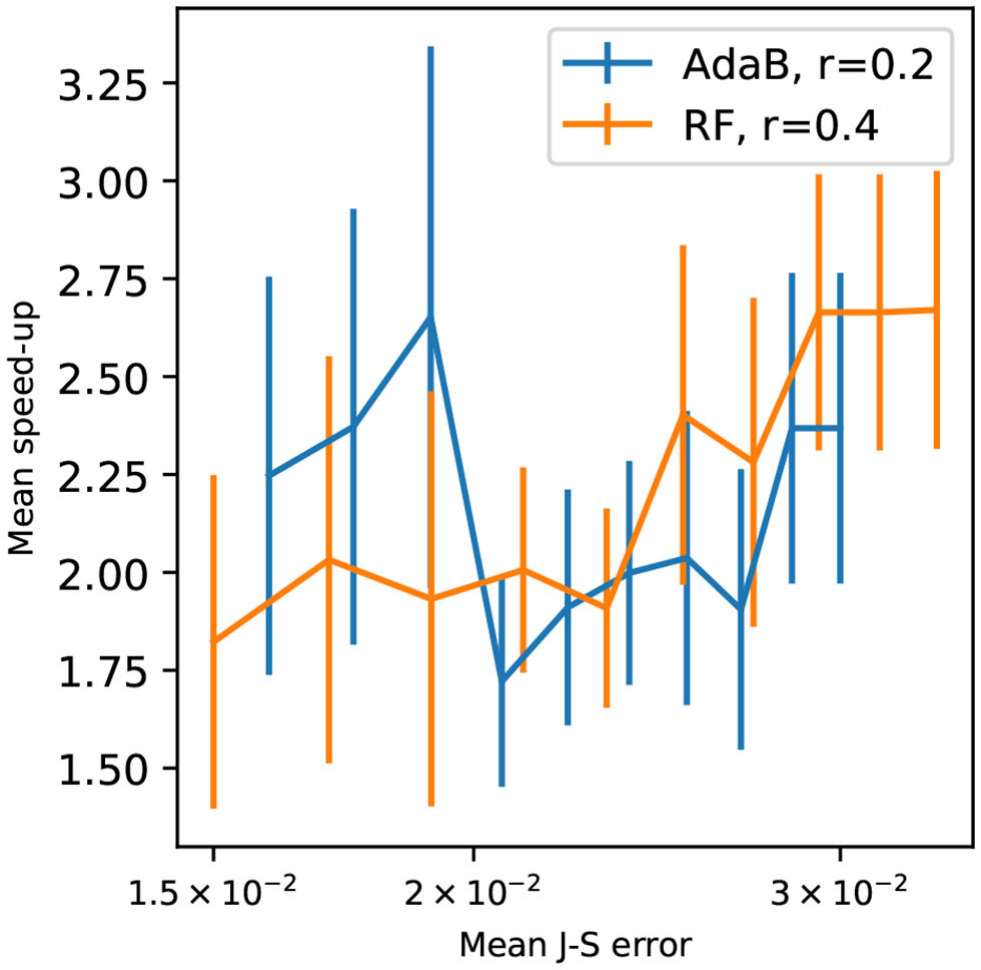
Speed-up with respect to the mean Jensen-Shannon error standard PoPEx : for RF-accelerated PoPEx with *r* = 0.4, and AdaBoost-accelerated with *r* = 0.2. Each point is an average over 4 PoPEx runs, and bars represent the standard error of the mean.

Figure 12 presents speed-up for different mean J-S errors. The speed-up was calculated as follows. It is the ratio of the computing time of the standard PoPEx algorithm by the computing time of ML-accelerated PoPEx for obtaining the same mean J-S errors. The simulation time was counted from iteration 500 to the iteration by which a specified mean J-S error was achieved. ML-accelerated modes achieve peak speed-ups greater than 2x for large errors, that is at the beginning of inversion procedure. The speed-ups for both ratios are similar and the *s-score* estimates well the real speed-up rate for *r* = 0.4. For *r* = 0.2 the *s-score* overestimated the speed-up.

## 5. Discussion and conclusion

In this article, we introduced a generic framework for accelerating the posterior population expansion algorithm (PoPEx) using machine learning techniques, and tested it on a groundwater transport problem. Our approach is generic and does not interfere with the geostatistical method used for modeling the prior and can be adapted to any type of forward problem. We found that in our set up, both Random Forest and AdaBoost performed well for classifying if generated models are of significance. While AdaBoost and Random Forest performed similarly for the two ratios, we recommend labeling a relatively high ratio of models as significant based on their likelihood—the training suffers from lower variations and results in more stable models. The obtained speedups are close to 2 for this problem, which can represent a significant gain of computing time and resources for large problems.

Random Forest and AdaBoost performance were comparable and superior to CNN in the classification task. Kelleher et al. (2015) pointed out that Random Forests perform well when the input has many features, as is the case in our study; AdaBoost performed well in the imbalanced case. The poor performance of CNN is surprising, as it benefits from information about the spatial arrangement of the input data. However, while CNN is very effective in recognizing shapes which are invariant by translation or rotation, in our specific case the absolute position of the features is important to predict the solute transport processes. The fact that simplified CNN architectures (which were reduced to feed-forward neural nets) performed generally better than the more complicated architectures, supports this claim. Further research is needed to determine if it is possible to design a specific Neural Network architecture outperforming the methods presented in this paper. Another important aspect is that the training dataset including only 500 elements is probably too small for the neural network to achieve good performance.

Another challenge for the ML methods is that the labels are arbitrary and depend on the relative values of the likelihood. The generated models are not “good” or “bad” in an absolute sense; they are “better” or “worse” than the others. It is possible that the same model receives different labels depending on other models in the ensemble. The imbalanced nature of the dataset makes it harder to train, especially for low values of the ratio *r*. We therefore recommend using the ratio *r* = 0.4, as it makes training easier and the performance of the classifier is more robust. Lower ratios are attractive not only from the point of view of the estimated speed-up, but also because lower ratios correspond to fewer good models, which is more relevant in terms of likelihood statistics (few models represent the majority of posterior weights). However, lower ratios suffer from the discrepancy between the estimated speed-up and the actual speed-up. This observation can be explained by the fact that rejecting a good model slows down the convergence of PoPEx. If a classifier missclassifies a good model, the core PoPEx algorithm misses an important information which could guide the sampling. PoPEx then continues sampling from a less-informed distribution, proposing worse models. This is why using a classifier with a low recall (low ratio value) is more penalizing than when using one with a higher recall (higher ratio values).

The proposed classification method could be employed in other Monte Carlo schemes, such as rejection sampling, importance sampling, or Metropolis algorithm (Tarantola, 2005). It could even yield better results than with PoPEx algorithm, as PoPEx is iteratively expanding the ensemble of models by learning from the already sampled models. Simpler samplers without memory of previously generated models could benefit more from the learning schemes.

Finally, an interesting alternative to using classifiers could be to use regression techniques to directly estimate the likelihood value. However, this would be a challenging task, as wrong likelihood estimations could mislead the sampler. It could even lead to slowing it down if irrelevant models would receive erroneously high likelihood. In the present work, erroneous classifications only slow down the inversion but they are not a source of noise.

## Data Availability Statement

The data presented in this study and accompanying code can be found below: https://doi.org/10.5281/zenodo.4574602.

## Author Contributions

PJ designed and implemented the methods, carried out all the coding and numerical experiments, and wrote the paper that was edited by PR. PR supervised the work.

## Conflict of Interest

The authors declare that the research was conducted in the absence of any commercial or financial relationships that could be construed as a potential conflict of interest.
